# Effects of health insurance integration on health care utilization and its equity among the mid-aged and elderly: evidence from China

**DOI:** 10.1186/s12939-019-1068-1

**Published:** 2019-10-29

**Authors:** Chaofan Li, Chengxiang Tang, Haipeng Wang

**Affiliations:** 1 Graduate School at Shenzhen, Tsinghua University, Shenzhen, 518000 China; 2 School of Public Administration, Guangzhou University, Guangzhou, 510006 China; 3 School of Health Care Management, Shandong University, Jinan, 250012 China; 4 NHC Key Laboratory of Health Economics and Policy Research (Shandong University), Shandong University, Jinan, 250012 China

**Keywords:** Health insurance integration, Health care utilization, Equity, Policy evaluation

## Abstract

**Background:**

The fragmentation of health insurance schemes in China has undermined equity in access to health care. To achieve universal health coverage by 2020, the Chinese government has decided to consolidate three basic medical insurance schemes. This study aims to evaluate the effects of integrating Urban and Rural Residents Basic Medical Insurance schemes on health care utilization and its equity in China.

**Methods:**

The data for the years before (2013) and after (2015) the integration were obtained from the China Health and Retirement Longitudinal Study. Respondents in pilot provinces were considered as the treatment group, and those in other provinces were the control group. Difference-in-difference method was used to examine integration effects on probability and frequency of health care visits. Subgroup analysis across regions of residence (urban/rural) and income groups and concentration index were used to examine effects on equity in utilization.

**Results:**

The integration had no significant effects on probability of outpatient visits (β = 0.01, *P* > 0.05), inpatient visits (β = 0.01, *P* > 0.05), and unmet hospitalization needs (β =0.01, *P* > 0.05), while it had significant and positive effects on number of outpatient visits (β = 0.62, *P* < 0.05) and inpatient visits (β = 0.39, *P* < 0.01). Moreover, the integration had significant and positive effects on number of outpatient visits (β = 0.77, *P* < 0.05) and inpatient visits (β = 0.49, *P* < 0.01) for rural residents but no significant effects for urban residents. Furthermore, the integration led to an increase in the frequency of inpatient care utilization for the poor (β = 0.78, *P* < 0.05) among the piloted provinces but had no significant effects for the rich (β = 0.25, *P* > 0.05). The concentration index for frequency of inpatient visits turned into negative direction in integration group, while that in control group increased by 0.011.

**Conclusions:**

The findings suggest that the integration of fragmented health insurance schemes could promote access to and improve equity in health care utilization. Successful experiences of consolidating health insurance schemes in pilot provinces can provide valuable lessons for other provinces in China and other countries with similar fragmented schemes.

## Introduction

Universal health coverage (UHC) is defined as “access to key promotive, preventive, curative and rehabilitative health interventions for all at an affordable cost, thereby achieving equity in access” by the 2005 World Health Assembly [1]. To achieve UHC is the goal of health system in each country. Health insurance, as a financial mechanism for UHC and health system, is widely used to promote equal access to health care utilization and financial protection worldwide [2–4]. The expansion of social health insurance coverage is widely considered as the important step towards achieving the goal of UHC that caters for everyone by providing access to adequate health services at an affordable price [5].

The Chinese government has committed to achieving UHC by 2020. To move toward UHC, the Chinese government has launched comprehensive social health insurance schemes since 1998. Three main schemes have been established to cover different socioeconomic groups, including Urban Employee Basic Medical Insurance for employees, New Rural Cooperative Medical Schemes (NRCMS) for farmers and Urban Residents Basic Medical Insurance (URBMI) for unemployed urban residents and children. The basic information of three health insurance schemes was displayed in Table 6 in Appendix. China successfully achieved universal health insurance in 2011, covering approximately 1.27 billion people (97% of the total population) [6]. About 2.1% of the mid-aged and elderly were covered by government medical insurance (GMI), and 0.9% were not covered by any kinds of health insurance schemes [7]. The rapid development of such schemes not only improved access to health care, but also provided financial protection to the entire population [8–10].

However, the fragmentation of health insurance schemes creates barriers for equal access to health care. The aforementioned three health insurance schemes are administrated by different departments and operated at municipal or county levels. The administrative departments can formulate financial and reimbursement policies according to local economic conditions and financing capacity. Owing to the differences in financial mechanisms and funding sources, substantial variations exist in benefit packages and reimbursement rates across enrollers with different insurance programs [6]. Rural enrollees have more limited benefit packages and lower reimbursement rates than those in urban regions. Thus, rural residents have more restricted access and financial barriers to utilizing health care than do urban residents. The systematic disparity in financial level and reimbursement policies across the schemes has resulted in unequal access to health care and financial protection [11, 12]. Moreover, the funding pools of different health insurance schemes and different districts are operated separately, which hinders risk and income subsidies across socioeconomic groups. The fragmentation of funding pools has also resulted in inequitable access to basic health care [13].

The experience of other countries shows that consolidation of health insurance schemes or financing mechanisms is a crucial strategy to promote equitable access to health care [14–17]. To improve equality in health care utilization between urban and rural residents, the Chinese government decided to merge NRCMS and URBMI to establish a unified medical insurance scheme for urban and rural residents, named as Urban-Rural Residents Basic Medical Insurance (URRBMI). Eight provinces piloted the integration of NRCMS and URBMI in 2014, while 23 provinces on the mainland have continued with the existing fragmented schemes [18]. The main integrating policies included unification of population coverage, fund pools, service packages, medical insurance drug lists, and reimbursement rates [19]. Compared with NRCMS, URRBMI has a more comprehensive service package, more drugs covered, and higher reimbursement rates [16, 20, 21]. The implementation of this integration is expected to narrow the gap between urban and rural residents with regards to access to health care. Thus, the impact of integration on health care utilization has become the focus of policymakers and researchers [5].

Previous studies have demonstrated that the differences in benefit packages and reimbursement rates between NRCMS and URBMI might lead to disparities in health care utilization [11, 22], and changes in benefit packages or reimbursement rates would affect the possibility of health services utilization and number of people using these services [8, 23–25]. Several case studies have described the practice, experiences, and challenges of consolidating NRCMS and URBMI in pilot areas [20, 21]. However, few studies have explored the impact of integration on health care utilization and distribution of impact across region of residence (urban/rural) or income groups. Reliable evidence on effects of consolidating medical insurance schemes is required if such integrating policies are to be extended throughout China and to provide policy implications to other countries with similarly fragmented health insurance schemes. The integration in the pilot provinces in 2014 provides an opportunity to evaluate the effects of this integration policy using a quasi-experiment design. Thus, this study aims to evaluate the effects of integrating NRCMS and URBMI on health care utilization and its equity using difference-in-differences (DID) analysis.

## Methods

### Data

The data were obtained from the China Health and Retirement Longitudinal Study (CHARLS), a nationally representative survey that includes 150 counties/districts from 28 provinces in China mainland [26]. Households and residents aged 45 years and older were sampled through multistage probability sampling, with follow-up surveys conducted every 2 years. The response rates were 82.63% in 2013 and 82.13% in 2015, respectively. The survey questionnaire covered a wide range of topics, including demographic characteristics, self-reported and objective physical health status, mental health, health functioning, insurance coverage, health-related behavior and health care utilization, work and retirement, economic conditions. All the sampled participants were interviewed by trained interviewers using a face-to-face computer-assisted personal interviewing system.

### Study design

**Fig. 1:**
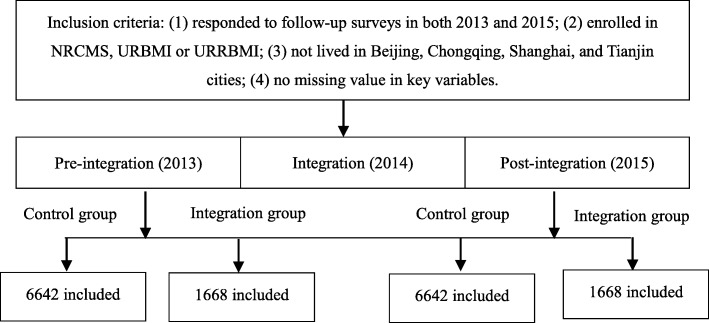
Flow chart of sample selection

A quasi-experimental design was used to ascertain the effects of medical insurance integration on health care utilization, and this is a powerful research design to study the causal impact of intervention in public health settings [27–29]. Excluding the super cities with high level of economic development (Beijing, Shanghai, Chongqing and Tianjin) or provinces not covered by CHARLS (Ningxia, Tibet and Hainan), respondents living in Zhejiang, Guangdong, Shandong, and Qinghai provinces comprised the integration group, while those residing in the other 20 provinces constituted the control group. Data from waves 2013 and 2015 were considered as pre-integration and post-integration, respectively. The inclusion criteria of the participants were: (1) responded to follow-up surveys in both 2013 and 2015; (2) enrolled in NRCMS, URBMI or URRBMI; (3) not lived in Beijing, Chongqing, Shanghai, and Tianjin cities; (4) no missing value in key variables. Ultimately, 8310 respondents were selected, with 6642 and 1668 in the control group and integration group, respectively. The detailed sampling process was shown in Fig. 1.

### Dependent variables

The key outcome variables for health care utilization were probability and number of outpatient visits in the previous month, probability and number of inpatient visits in the previous year, and unmet hospitalization need. The probability of outpatient visit was based on the question: “In the past month, have you visited a public hospital, private hospital, public health center, clinic, or health worker’s or doctor’s practice, or been visited by a health worker or doctor for outpatient care”. The number of outpatient visits was the total number of visits of the respondents in the previous month. Probability of inpatient visits was based on the question “Have you received inpatient care in the past year”, and the number of inpatient visits was based on the question “How many times have you received inpatient care during the past year”. Unmet hospitalization need was based on the question “In the past year, did you choose not to go to hospital after a doctor had suggested that you needed inpatient care”. Answers on probability of outpatient visit, inpatient visit and unmet hospitalization need were coded as dichotomous variables: 0, no; 1, yes. Answers on number of outpatient or inpatient visits were coded as count variables.

### Control variables

According to Andersen’s behavior model, the control variables consisted of predisposing factors, enabling factors and need factors, which were used to adjust for health care utilization [30–32]. Predisposing factors included gender (0, female; 1, male), age, education level (1, lower than primary school; 2, primary school; 3, middle school; 4, high school and above), marital status (0, married or partnered; 1, separated, divorced and widowed), and occupation status (1, agricultural work; 2, employed; 3, self-employed; 4, unemployed or retired). Enabling factors included region or residence (0, urban community; 1, rural village) and economic status. The region of residence was divided into urban and rural areas, which differed in access to transportation and medical facilities [31]. Economic status was measured by per capita household expenditure and evenly divided into three groups (1, poor; 2, medium; 3, rich). Need variables included self-reported health status (1, very good and good; 2, fair; 3, poor and very poor) and presence of chronic diseases (0, no; 1, yes).

### Statistical analysis

Descriptive analysis was conducted to summarize the basic characteristics of the selected respondents. Chi-square test was used to examine differences in probabilities of health care utilization between before and after the integration. Kruskal-Wallis rank test was conducted to examine differences in number of health care visits which usually had right-skewed distribution. DID analysis was used to capture the effects of medical insurance schemes’ integration on health care utilization as follows:

1 $$ {y}_{it}={\beta}_0+{\beta}_1{G}_i+{\beta}_2{P}_t+{\beta}_3{G}_i\times {P}_t+\gamma {X}_{it}+{\varepsilon}_{it} $$

where *y*_*it*_ is the outcome variable of health care utilization of respondent *i* at time *t*; *G*_*i*_ is the integration dummy variable that equals 1 if the respondent is in the provinces which integrated NRCMS and URBMI and 0 if in the control group which did not integrated health insurance schemes; *P*_*t*_ is the year dummy variable that equals 0 denoting time before NRCMS and URBMI integration (the year of 2013) and 1 denoting the time after integration (the year of 2015), respectively; *G*_*i*_ × *P*_*t*_ is the interaction of the integration dummy variable and year dummy variable, and *β*_3_ captures the average treatment effect of integration on health care utilization among the treatment group; *X*_*it*_ is a set of covariates of resident *i* at time *t*. We first conducted DID analysis without covariates, and then performed DID analysis with covariates to capture control variable adjusted results. Furthermore, we performed subgroup analysis across region of residence (urban/rural) and income groups to obtain the distribution of treatment effects. Multivariate logistic regression was used to estimate for binary outcome variables and negative binomial regression was used to estimate for count outcome variables.

To examine the change of equity in health care utilization before and after URRBMI integration, we also calculated concentration index for each outcome variable to compare them. A “convenient covariance” method was used to compute the concentration index:

2 $$ \mathrm{C}=\frac{2}{\mu}\mathit{\operatorname{cov}}\left(y,r\right) $$

in which C denotes concentration index, y is the health care utilization variable, μis its mean, r is the rank of economic status variable from poorest to richest, and cov is the covariance between health care utilization variable and fractional rank of economic status [33]. The value of concentration index ranges from − 1 to 1: positive value indicates that health care utilization is disproportionately concentrated among the rich, while negative value means health care utilization is higher among the poor, zero means health care utilization is evenly distributed across various income groups. We used Stata 15.1 for all the statistical analyses.

## Results

Table 1 shows the basic characteristics of the sample. Most participants were female (54%), married or with partners (90%), living in rural areas (73%), and low educated (47%). More than half of the participants engaged in agricultural work, and about one-fifth of them were unemployed or retired. More than 20% reported their health status as very good or good, while about 25% assessed their health status as poor or very poor. More than 60% of the participants had at least one chronic disease. Per capita household expenditure of the control group was higher than that of the treatment group.

**Table 1:** Basic characteristics of participants by time and integration policy, *N* (%)

Variables	2013		2015	
	Control (*N* = 6642)	Integration (*N* = 1668)	Control (*N* = 6642)	Integration (*N* = 1668)
Gender				
Female	3560 (53.60)	898 (53.84)	3560 (53.60)	898 (53.84)
Male	3082 (46.40)	770 (46.16)	3082 (46.40)	770 (46.16)
Age, years (Mean, SD)	58.36 (9.03)	58.63 (9.09)	60.31 (9.04)	60.51 (9.02)
Education				
Lower than primary school	3168 (47.70)	790 (47.36)	3168 (47.70)	790 (47.36)
Primary school	1603 (24.13)	420 (25.18)	1603 (24.13)	420 (25.18)
Middle school	1417 (21.33)	339 (20.32)	1417 (21.33)	339 (20.32)
High school and above	454 (6.84)	119 (7.13)	454 (6.84)	119 (7.13)
Marital status				
Married or partnered	5984 (90.09)	1495 (89.63)	5866 (88.32)	1464 (87.77)
Separated, divorced and widowed	658 (9.91)	173 (10.37)	776 (11.68)	204 (12.23)
Occupation status				
Agricultural work	3980 (59.92)	830 (49.76)	3608 (54.32)	766 (45.92)
Employed	652 (9.82)	309 (18.53)	793 (11.94)	340 (20.38)
Self-employed	604 (9.09)	152 (9.11)	505 (7.60)	129 (7.73)
Unemployed or retired	1406 (21.17)	377 (22.60)	1736 (26.14)	433 (25.96)
Region of residence				
Urban community	1802 (27.13)	525 (31.47)	1802 (27.13)	525 (31.47)
Rural village	4840 (72.87)	1143 (68.53)	4840 (72.87)	1143 (68.53)
Per capita household expenditure^a^ (Mean, SD)	9822.32 (16,202.46)	9059.274 (11,266.19)	11,672.30 (17,702.85)	10,925.49 (13,360.13)
Self-reported health status				
Very good and good	1409 (21.21)	544 (32.61)	1457 (21.94)	472 (28.30)
Fair	3576 (53.84)	830 (49.76)	3528 (53.12)	900 (53.96)
Poor and very poor	1657 (24.95)	294 (17.63)	1657 (24.95)	296 (17.75)
Presence of chronic disease				
No	1758 (26.47)	630 (37.77)	1688 (25.41)	579 (34.71)
Yes	4884 (73.53)	1038 (62.23)	4954 (74.59)	1089 (65.29)

*Note*: ^a^ The unit of the annual per capita household expenditure is Chinese Yuan

Table 2 presents changes in health care utilization of respondents before and after health insurance scheme integration. From 2013 to 2015, the probability of outpatient visit decreased from 19.7 to 17.7% for the control group, and from 16.4 to 15.2% for the treatment group. The average number of outpatient visits for the control group reduced 0.11 times, while it increased 0.50 times for the treatment group. The probability of inpatient visit for both groups increased, but it increased more for the treatment group (1.9%) than for the control group (1.1%). Moreover, the average number of inpatient visits decreased 0.02 times for the control group, while it increased 0.36 times for the treatment group. The probability of unmet hospitalization need decreased by 0.4% for the control group, but increased by 0.6% for the treatment group.

**Table 2:** Health care utilization before and after Urban-rural Residents Basic Medical Insurance Integration

Variables	Control			Integration		
	2013	2015	D1	2013	2015	D2
Probability of outpatient visit last month (%)						
All	19.65	17.65	−2.00^***^	16.43	15.17	−1.26
Urban community	18.98	16.20	−2.78^**^	17.71	16.57	−1.14
Rural village	19.90	18.18	−1.72^**^	15.84	14.52	−1.32
Poor	18.76	17.40	−1.36	14.12	13.95	−0.17
Medium	19.51	17.97	−1.54	17.61	14.50	−3.11
Rich	20.63	17.56	−3.07^***^	17.85	17.27	−0.58
Number of outpatient visits last month (Mean)						
All	2.33	2.22	−0.11	2.21	2.71	0.50
Urban community	2.33	2.09	−0.24	2.41	2.54	0.13
Rural village	2.33	2.26	−0.07	2.10	2.80	0.70
Poor	2.46	2.25	−0.21	2.20	2.96	0.77
Medium	2.18	2.24	0.06	1.79	2.39	0.60
Rich	2.37	2.18	−0.19	2.65	2.76	0.11
Probability of inpatient visit last year (%)						
All	11.34	12.42	1.08^*^	7.01	8.93	1.92^**^
Urban community	12.54	12.60	0.06	8.00	9.52	1.52
Rural village	10.89	12.36	1.47^**^	6.56	8.66	2.10^*^
Poor	9.22	9.88	0.66	5.32	6.98	1.66
Medium	11.65	12.17	0.52	8.81	6.42	−2.39
Rich	13.07	15.12	2.05^**^	7.10	13.82	6.72^***^
Number of inpatient visits last year (Mean)						
All	1.49	1.47	−0.02	1.24	1.60	0.36^*^
Urban community	1.50	1.41	− 0.09	1.36	1.48	0.12
Rural village	1.49	1.49	0.00	1.17	1.66	0.49^***^
Poor	1.54	1.47	−0.07	1.13	1.83	0.71
Medium	1.50	1.40	−0.10	1.33	1.46	0.12
Rich	1.46	1.52	0.06	1.22	1.53	0.31
Probability of unmet hospitalization need last year (%)						
All	6.27	5.90	−0.37	3.03	3.66	0.63
Urban community	6.07	5.61	−0.46	2.43	4.19	1.76
Rural village	6.34	6.01	−0.33	3.25	3.41	0.16
Poor	6.02	5.35	−0.67	2.72	3.65	0.93
Medium	7.07	6.38	− 0.69	2.65	4.04	1.39
Rich	5.73	5.96	0.23	3.83	3.26	−0.57

*Note*: D1, change in health service utilization during the period of pre- and post- integration in the control group; D2, change in the integration group

^*^, *P* < 0.1; ^**^, *P* < 0.05; ^***^, *P* < 0.01

Table 3 displays the effects of integration of medical insurance schemes on health care utilization. In terms of the probability of outpatient visit, the treatment effect of integration was not significant (β = 0.01, *P* > 0.1). In contrast, the integration had positive effect on number of outpatient visits (β = 0.62, *P* < 0.05). The coefficients of interaction terms for probability and number of inpatient care visits were 0.01 (*P* > 0.1) and 0.39 (*P* < 0.01), respectively. These results mean that the integration also had positive treatment effects on the frequency of inpatient care utilization. As regards the unmet hospitalization need, the treatment effect of integration was an increase in probability (β = 0.01), but not significant.

**Table 3:** The effects of Urban-rural Residents Basic Medical Insurance Integration on health care utilization

Variables	DID without covariates		DID with covariates	
	β	95% CI	β	95% CI
Probability of outpatient visit last month				
year 2015	−0.02^***^	(− 0.03, − 0.01)	−0.02^***^	(− 0.0,3–0.01)
Integration	− 0.03^***^	(− 0.05, − 0.01)	−0.01	(− 0.02, 0.01)
year 2015 × Integration	0.01	(− 0.02, 0.04)	0	(−0.03, 0.03)
Number of outpatients visits last month				
year 2015	−0.11	(− 0.28, 0.06)	− 0.10	(− 0.27, 0.07)
Integration	− 0.13	(− 0.43, 0.18)	− 0.02	(− 0.32, 0.28)
year 2015 × Integration	0.62^**^	(0.05, 1.18)	0.59^**^	(0.02, 1.15)
Probability of inpatient visit last year				
year 2015	0.01^*^	(0, 0.02)	0	(−0.01, 0.01)
Integration	− 0.04^***^	(− 0.06, − 0.03)	−0.03^***^	(− 0.04, − 0.01)
year 2015 × Integration	0.01	(− 0.01, 0.03)	0	(− 0.02, 0.03)
Number of inpatients last year				
year 2015	−0.03	(− 0.12, 0.07)	− 0.07	(− 0.16, 0.02)
Integration	− 0.25^***^	(− 0.38, − 0.13)	−0.22^***^	(− 0.34, − 0.09)
year 2015 × Integration	0.39^***^	(0.12, 0.65)	0.36^***^	(0.09, 0.62)
Probability of unmet hospitalization needs last year				
year 2015	0	(−0.01, 0.01)	0	(−0.01, 0.01)
Integration	−0.03^***^	(−0.04, − 0.02)	− 0.02^***^	(− 0.03, − 0.01)
year 2015 × Integration	0.01	(− 0.01, 0.03)	0.01	(− 0.01, 0.02)

*Note*: *β* coefficients, *CI* confidence interval, *DID* difference-in-differences

^*^, *P* < 0.1; ^**^, *P* < 0.05; ^***^, *P* < 0.01

Table 4 shows the effects of integration of medical insurance schemes on the number of outpatient and inpatient visits across region of residence and income groups. As regards number of outpatient visits, the coefficient of interaction term for the urban community and rural village were 0.37 (*P* > 0.1) and 0.77 (*P* < 0.05), respectively. This means that the integration of medical insurance schemes had positive treatment effects on the frequency of outpatient care utilization for rural enrollees, but no significant effects for urban enrollees. Moreover, the coefficients of interaction terms for the number of inpatient visits were 0.21 (*P* > 0.1) and 0.49 (*P* < 0.01), respectively. This result also means that the integration had positive effects on the number of inpatient visits for rural residents but no effects for urban residents. Furthermore, the coefficient of interaction terms for number inpatient visits among the poor was 0.78 (*P* < 0.05), while them among the medium and rich were positive but rarely significant. It means that the integration had positive effects on the number of inpatient visits for the poor residents but no effects for the medium and rich residents.

**Table 4:** The effects of URRBMI Integration on frequency of health care utilization across subgroups

Subgroup	Variables	DID without covariates		DID with covariates	
		β	95% CI	β	95% CI
Number of outpatients visits last month					
Urban	year 2015	− 0.24	(− 0.59, 0.12)	−0.24	(− 0.59, 0.11)
	Integration	0.08	(−0.52, 0.69)	0.14	(−0.46, 0.74)
	year 2015 × Integration	0.37	(−0.51, 1.25)	0.34	(−0.55, 1.23)
Rural	year 2015	−0.07	(−0.27, 0.13)	− 0.05	(− 0.25, 0.15)
	Integration	−0.24	(− 0.58, 0.11)	− 0.10	(− 0.43, 0.24)
	year 2015 × Integration	0.77^**^	(0.04, 1.51)	0.73^**^	(0, 1.46)
Poor	year 2015	−0.21	(− 0.53, 0.11)	− 0.19	(− 0.50, 0.13)
	Integration	− 0.26	(− 0.76, 0.24)	− 0.18	(− 0.68, 0.32)
	year 2015 × Integration	0.98	(− 0.20, 2.16)	0.96	(− 0.23, 2.15)
Medium	year 2015	0.06	(−0.24, 0.35)	0.06	(−0.24, 0.35)
	Integration	−0.39^**^	(−0.77, − 0.01)	−0.19	(− 0.57, 0.19)
	year 2015 × Integration	0.54^*^	(−0.10, 1.19)	0.46	(−0.17, 1.10)
Rich	year 2015	−0.19	(−0.47, 0.09)	− 0.17	(− 0.45, 0.11)
	Integration	0.28	(−0.37, 0.93)	0.32	(−0.32, 0.96)
	year 2015 × Integration	0.30	(−0.72, 1.31)	0.36	(−0.65, 1.36)
Number of inpatients visits last year					
Urban	year 2015	−0.09	(−0.26, 0.08)	− 0.11	(− 0.27, 0.05)
	Integration	−0.14	(− 0.37, 0.08)	−0.06	(− 0.27, 0.16)
	year 2015 × Integration	0.21	(−0.24, 0.66)	0.11	(−0.35, 0.56)
Rural	year 2015	0	(−0.11, 0.11)	−0.05	(− 0.16, 0.06)
	Integration	−0.32^***^	(−0.46, − 0.17)	−0.30^***^	(− 0.45, − 0.15)
	year 2015 × Integration	0.49^***^	(0.16, 0.81)	0.48^***^	(0.16, 0.81)
Poor	year 2015	−0.07	(− 0.25, 0.11)	−0.07	(− 0.25, 0.11)
	Integration	−0.41^***^	(−0.62, − 0.20)	−0.36^***^	(− 0.57, − 0.16)
	year 2015 × Integration	0.78^**^	(0.08, 1.47)	0.74^**^	(0.03, 1.46)
Medium	year 2015	−0.10	(− 0.27, 0.06)	−0.15^*^	(− 0.31, 0.01)
	Integration	−0.17	(−0.41, 0.07)	− 0.09	(− 0.33, 0.15)
	year 2015 × Integration	0.23	(−0.14, 0.59)	0.13	(−0.21, 0.48)
Rich	year 2015	0.06	(−0.08, 0.21)	0	(−0.13, 0.14)
	Integration	−0.24^***^	(−0.41, − 0.07)	−0.26^***^	(− 0.43, − 0.10)
	year 2015 × Integration	0.25	(−0.06, 0.56)	0.27^*^	(−0.03, 0.58)

*Note*: *β* coefficients, *CI* confidence interval, *DID* difference-in-differences, *URRBMI* Urban-rural Residents Medical Insurance

^*^, *P* < 0.1; ^**^, *P* < 0.05; ^***^, *P* < 0.01

Table 5 displays the change of concentration index in health care utilization between before and after URRBMI integration. In the case of probability of outpatient and inpatient visits, the concentration index were significantly positive (favoring the rich) in both control and integration groups and both before and after the integration of URRBMI. This implied that the integration of URRBMI had little impacts on equity in probability of health care utilization. In contrast, the concentration index for frequency of inpatient visits changed into negative direction (favoring the poor) in integration group, while that in control group increased by 0.011. This illustrated that the integration policy had reduced the inequality in frequency of inpatient care utilization. The concentration index probability of unmet hospitalization need remained positive in both control and integration groups after integration, which implied that the integration policy had little effects on equity in unmet hospitalization need.

**Table 5:** The changes of concentration index in health care utilization before and after URRBMI integration

Variables	Control		Integration	
	2013	2015	2013	2015
Probability of outpatient visit last month	0.031^**^	0.006	0.058^*^	0.115^**^
Number of outpatient visits last month	0.010	−0.006	0.006	0.000
Probability of inpatient visit last year	0.122^***^	0.126^***^	0.070	0.189^***^
Number of inpatient visits last year	0.002	0.013	−0.006	− 0.004
Probability of unmet hospitalization need last year	0.013	0.040	−0.035	0.049

*Note*: *URRBMI* Urban-rural Residents Medical Insurance

^*^, *P* < 0.1; ^**^, *P* < 0.05; ^***^, *P* < 0.01

## Discussion

This study provides evidence on the positive treatment effects of integration NRCMS and URBMI on health care utilization. We not only examine the effects of integration on probability and frequency of health care utilization, but also evaluate its impact on equality in utilization. The findings imply that the integration plays an important role in moving China towards achieving UHC. The integration improved the scale and depth of coverage and obviously increased the number of outpatient and inpatient care visits, although it had no significant effects on probability of health care utilization and unmet hospitalization need. Moreover, the subgroup analysis demonstrates that the frequency of health care visits increased much more among rural residents than that among urban residents, and increased more among poor residents than that among rich residents. This finding implies that the integration narrowed the gaps in health care utilization not only between rural and urban areas, but also between lowest-income group and highest-income group. Thus, the integration improved equitable access to health care.

We find that the integration of NRCMS and URBMI increased the frequency of health care utilization in the integration group. Two reasons might explain this positive effect: First, the integration extended the service package and drug list coverage, which improved access to health care for the enrollees, especially rural residents enrolled in NRCMS with a limited benefit package before 2014. Previous studies have demonstrated that the extended benefit package of health insurance increases the frequency of health care visits [23, 34, 35]. Second, the integration increased reimbursement rates of health expenditure, which improved the affordability of health care. The literature indicates that changes in reimbursement rates decreased the expenditure for both outpatient and inpatient services and increased the number of health care visits for enrollees [24, 25]. Financial access can be improved by reducing out-of-pocket (OOP) payments through insurance prepayments [36]. This study demonstrates that health insurance integration promoted the depth (the inclusion of all needed services) and height (the proportion of costs covered) of UHC through expanding the benefit package and increasing reimbursement rates.

However, the probability of health care utilization did not significantly increase and the probability of unmet hospitalization need remained high after the integration. A possible explanation is that the deductibles of the URRBMI and OOP for both outpatient and inpatient services are somewhat high, which are the main barriers for the poor to initiate health care visits [37]. Although the integration extended the benefit package and increased the reimbursement rate, it did not decrease the OOP expenditure for inpatient visits and the deductibles persisted. The increase in total heath expenditure was more than the increase in the reimbursement of medical insurance and the integration had no effects on OOP for inpatient expenditure [20]. Thus, the poor still had to face high deductibles and bear the high copayment expenditure for inpatient care, which led to financial difficulty in health care utilization. Hence, a high proportion of enrollees did not utilize inpatient care when they required it. This finding implies that more attention should be paid to the unmet need of inpatient services among the poor. Therefore, to ensure access to basic health care for all Chinese citizens, we should focus first on increasing its coverage and decreasing economic barriers to access among the disadvantaged populations in the process of health insurance consolidation [38, 39].

This study also finds that the integration of the NRCMS and URBMI had heterogeneous effects on health care utilization between urban and rural residents. First, it promoted the rural enrollees to have a larger increment of outpatient and inpatient service utilization, while it had no positive effects among the urban enrollees. This discrepancy is mainly because the integration had extended the benefit package and increased the reimbursement rate, and rural and urban residents were covered by the same benefit packages and reimbursement policies [19]. Compared with urban residents, rural residents experienced greater improvement in terms of service package, drug coverage and reimbursement rates after the integration [16]. Further, no significant difference in health care utilization was observed between urban and rural enrollees after controlling for other factors (e.g., age and health status), which means that the integration reduced urban-rural disparity in health care utilization. Second, the low-income groups may benefit more from the integration. The integration policy had positive treatment effects on frequency of inpatient care utilization for the poor, while no significant effects for the medium and rich. This finding was consistent with other studies that estimated the effects of extending health insurance coverage or extending benefit coverage [34, 40, 41]. The possible explanation is that the low-income individuals are more sensitive to cost-sharing and have a higher elasticity of health care than the high-income group [42, 43]. Even if facing the same cost, the low-income populations have to pay higher proportion of income for the health care than the high-income individuals [35]. If benefit coverage extends or copayment decreases, the poor may have larger increase in health care utilization than the rich, so the inequality reduced; and vice versa [35, 44, 45].

This study has several limitations. First, the study period covers only 1 year each before and after the integration. The short study period may not reflect the long-term effects of integration of medical insurance schemes on health care utilization. The literature suggests that most nations required a long period to complete their UHC efforts via consolidating health insurance [12]. Second, this study focuses on health care utilization instead of health status improvement and financial protection, which is the final goal of health systems. Further research should be conducted to examine the effects of policy on health outcomes and catastrophic health expenditure. Third, the integration groups are mainly concentrated in China’s eastern provinces, which may bias the results and result in overestimation of the effects. Studies on the central and west provinces are needed to provide comprehensive evidence. However, this study employed a quasi-experiment study design and DID analysis, which could identify the treatment effects of the integration policy.

## Conclusions

In conclusion, this study demonstrates that positive effect exists between the integration of health insurance schemes and equity in health care utilization in China. The integration could both increase the frequency of health care utilization and improve equity in utilization. However, the integration had no significant effects on the probability of health care visits and unmet hospitalization need, which suggests that policymakers should pay more attention to the disadvantaged groups and implement more targeting measures to improve equitable access to health care. Overall, the integration of insurance schemes is imperative, feasible, and effective in China, which marks an important stride toward UHC. Successful experiences of integrating health insurance schemes in pilot provinces can provide valuable lessons on key issues for other provinces in China. This study may provide policy implications to other low- and middle-income countries that currently have fragmented health insurance schemes and aim to achieve UHC to ensure inherent equity for all, such as Vietnam, India, and Iran.

## Data Availability

The datasets generated and analyzed during the current study are available in the China Health and Retirement Longitudinal Study repository, [http://charls.pku.edu.cn/zh-CN].

## Appendix

We also conducted a propensity score matching with difference-in-differences analysis to estimate the effects of health insurance integration. Kernel-based propensity score matching with bandwidth 0.06 was performed, in which only 1 case from integration group and 37 cases from control group were excluded (8,310 cases in the whole sample). The analysis indicated that basic characteristics between integration group and control group were comparable after propensity score matching. The results from PSMDID analysis were robust. We put the results in the Table 7, 8 and 9 in appendix.

**Table 6:** Summary of three main health insurance schemes in China

Items	UEBMI	NCMS	URBMI
Inception year	1998	2003	2007
Administrator	MOHRSS	NHC	MOHRSS
Eligible population	Urban employees and retirees	Rural residents	Urban unemployed residents, students, children, etc.
Enrolment unit/type	Individual/mandatory	Family/voluntary	Individual/ voluntary
Number of enrollees in 2013 (millions)	296	802	274
Source of financing	payroll tax (6% from employers, and 2% from employee)	Individual contribution and government subsidy	Individual contribution and government subsidy
Per capita fund in 2013 (CNY)	2573.19	370.59	400.48
Pooling level	Municipal	County	Municipal
Service package	Comprehensive	Limited	Limited
Number of drugs covered	2300	800	2300
Whether covers outpatient care	Yes	70% covering, 30% not covering	No covering in principle
Inpatient compensation rate in 2013 ^a^	95.3	91.1	88.7
Inpatient reimbursement rate in 2013 (%)	68.8	50.1	53.6

*Note*: Data are from 2014 China Statistical Yearbook and An Analysis Report of National Health Services Survey in China, 2013

*UEBMI* Urban Employees Basic Medical Insurance, *NCMS* New Rural Cooperative Medical Schemes, *URBMI* Urban Residents Basic Medical Insurance, *MOHRSS* Ministry of Human Resources and Social Security, *NHC* National Health Commission, *CNY* Chinese Yuan

^a^, the proportion of the number of individuals obtaining reimbursement to the total number of individuals having inpatient service utilization

**Table 7:** Balancing test of covariates between the control and treated groups after propensity score match

Variable(s)	Mean Control	Mean Treated	Differences	t	*P*
Gender	0.46	0.46	0	0.08	0.94
Age	58.59	58.63	0.05	0.19	0.85
Educational level	1.87	1.87	0	0.02	0.98
Marital status	1.10	1.10	0	0.03	0.98
Occupational status	2.02	2.05	0.02	0.65	0.52
Region of residence	0.69	0.69	−0.01	0.56	0.57
Self-rated health status	1.88	1.85	−0.03	1.49	0.14
Presence of chronic disease	0.65	0.62	−0.03	1.89	0.06
Log of per capita consumption expenditure	8.78	8.78	−0.01	0.25	0.80

**Table 8:** The effects of Urban-rural Residents Medical Insurance Integration on probability of health care utilization using PSM-DID analysis

Variables	β	95% CI	
Outpatient visit last month			
year 2015	−0.01^*^	− 0.02	0
Integration	−0.01	−0.03	0.01
year 2015 × Integration	0	−0.03	0.03
Inpatient visit last year			
year 2015	0.01^**^	0	0.02
Integration	−0.03^***^	−0.04	− 0.01
year 2015 × Integration	0.01	−0.01	0.03
Unmet hospitalization needs last year			
year 2015	0	−0.01	0.01
Integration	−0.02^***^	−0.04	− 0.01
year 2015 × Integration	0	−0.01	0.02

*β* coefficients, *CI* confidence interval, *PSM-DID* propensity score matching combined with difference-in-differences

^*^, *P* < 0.1; ^**^, *P* < 0.05; ^***^, *P* < 0.01

**Table 9:** The effects of Urban-rural Residents Medical Insurance Integration on frequency of health care utilization using PSM-DID analysis

Variables	Number of outpatients visits last month			Number of inpatients last year		
	β	95% CI		β	95% CI	
Total population						
year 2015	0.17	−0.07	0.41	0.27^***^	0.10	0.43
Integration	−0.04	−0.35	0.27	−0.22^***^	− 0.35	−0.10
year 2015 × Integration	0.96^*^	−0.07	1.99	0.27	−0.30	0.85
Urban						
year 2015	−0.18	−0.60	0.24	0.44^*^	−0.01	0.90
Integration	0.10	−0.51	0.72	−0.11	−0.33	0.10
year 2015 × Integration	0.62	−0.91	2.16	−0.20	−1.12	0.73
Rural						
year 2015	0.27^*^	−0.02	0.55	0.26^**^	0.05	0.47
Integration	−0.13	−0.47	0.21	−0.32^***^	− 0.47	−0.16
year 2015 × Integration	1.29^*^	−0.07	2.66	0.43	−0.30	1.17
Poor						
year 2015	0.05	−0.38	0.49	0.22	−0.06	0.50
Integration	−0.25	−0.75	0.26	−0.33^***^	− 0.53	−0.13
year 2015 × Integration	2.59^*^	−0.08	5.26	1.46^*^	−0.20	3.11
Medium						
year 2015	0.26	−0.19	0.71	0.18	−0.16	0.52
Integration	−0.24	−0.62	0.14	−0.09	− 0.34	0.15
year 2015 × Integration	0.57	−0.47	1.61	0.02	−0.64	0.69
Rich						
year 2015	0.06	−0.34	0.45	0.41^***^	0.16	0.66
Integration	0.26	−0.40	0.91	−0.26^***^	− 0.43	−0.09
year 2015 × Integration	−0.08	−1.16	1.00	−0.18	−1.01	0.65

β, coefficients; CI, confidence interval; PSM-DID, propensity score matching combined with difference-in-differences

^*^, *P* < 0.1; ^**^, *P* < 0.05; ^***^, *P* < 0.01

## Abbreviations

CHARLS: China Health and Retirement Longitudinal Study
DID: Difference-in-differences
GMI: government medical insurance
NRCMS: New Rural Cooperative Medical Schemes
OOP: out-of-pocket
UHC: universal health coverage
URBMI: Urban Residents Basic Medical Insurance
URRBMI: Urban-Rural Residents Basic Medical Insurance
